# Sustainable employability for older workers: an explorative survey of belgian companies

**DOI:** 10.1186/s13690-016-0128-x

**Published:** 2016-04-28

**Authors:** Mathieu Verbrugghe, Yoline Kuipers, Bart Vriesacker, Ilse Peeters, Katrien Mortelmans

**Affiliations:** Mensura Occupational Health Services, Brussels, Belgium; Department of Public Health, Ghent University, Ghent, Belgium; Milieu Ltd - Law & Policy Consulting, Brussels, Belgium

**Keywords:** Sustainable employability, Belgium, Older workers, Occupational health, Collective labour agreement

## Abstract

**Background:**

The European Agency for Safety and Health at Work (EU-OSHA) is developing an online e-guide, which will provide tips and practical information for each EU country (in their national language(s)) on ageing and occupational health and safety. The e-guide will be launched in 2016 as part of the EU-OSHA campaign on Healthy Workplaces for all ages. The e-guide will present evidence, tools and practical examples of how companies can take action and effectively promote sustainable employability.

**Methods:**

As part of the development of the e-guide, a cross-sectional study was conducted to survey Belgian employers in April 2015 to determine their specific needs concerning older workers’ occupational health and safety issues. Researchers from Milieu Ltd. (Brussels, Belgium), the consultancy company coordinating the e-guide project, and Mensura Occupational Health Services (Brussels, Belgium) developed a 13-item questionnaire. The survey addressed the needs and importance given to sustainable employability of older workers in Belgian companies and evaluated corporate knowledge regarding relevant national policies. The questionnaire was distributed electronically to the management of 22,084 private-sector companies affiliated with Mensura.

**Results:**

Ten percent (*n* = 2133) of recipients opened the e-mail, and 37 % (*n* = 790) of these completed the questionnaire. In 89 % of the responding companies, sustainable employability of workers aged ≥55 years plays an important role; 70 % have no active sustainable employability policy/initiative; 18 % experience difficulties promoting sustainable employability; and 86 % indicate no need for support to promote sustainable employability. Respondents noted the following health complaints among workers aged ≥55 years: work-related health problems (31 %), stress (26 %), work agreements/type of work (17 %), work/life balance (15 %), and career development and/or training (9 %). Topics concerning health and well-being of workers aged ≥55 years requiring the most attention include motivation (30 %) and adaptation of the workplace to their health requirements (26 %).

**Conclusions:**

The e-guide should raise further awareness among employers about the importance of implementing an active sustainable employability policy to prolong working life in a healthy and productive way. The e-guide should also include tools to address work-related health problems and stress, motivation, and adaptation of the workplace to the health requirements of workers aged ≥55 years.

## Background

The population of the European Union (EU) is ageing as a result of increasing life expectancy and low birth rates [1]. Consequently, the workforce is ageing and the proportion of working people decreasing, which impacts social security systems. The EU employment rate for the population aged 55–64 years increased from 38.4 % in 2002 to 51.8 % in 2014 [1]. In Belgium, the employment rate for older individuals is lower than the EU average; in 2014, only 42.7 % of the Belgian population 55–64 years old was employed.

To address the current and future impact of the ageing population, it is important to increase employment levels of all workers—including levels of older workers. The goal is to prolong working life and ensure that older workers maintain good health so they are able to work until retirement age, or even extend their professional careers beyond that point. In Belgium, to counteract the low employment rates among workers aged ≥55 years, the federal government approved measures such as the Experience Fund, which offers financial support to companies with targeted initiatives to retain or improve work ability of older workers (*e.g.*, increasing employability by means of training and skills enhancement). Additionally, the government raised the official retirement age from 65 to 66 years by 2025 and to 67 by 2030.

Employers are challenged to keep ageing workers motivated and employable without threatening their well-being [2]. The concept of sustainable employability has received much attention in the last few years. Van der Klink et al. [3] defined sustainable employability as “*workers having the opportunity to perform work with preservation of good health and wellbeing in current and future working life”.*

In Belgium, a mandatory collective labour agreement (CLA 104) was introduced in 2013 to support the sustainable employability of workers aged ≥45 years. CLA 104 obligates companies in the private sector with more than 20 workers to develop and implement a concrete action plan to promote the employment of workers ≥45 years, *e.g.,* by introducing flexible work schedules, developing health promotion programs, and organizing customized trainings. Additionally, at the EU level, the European Agency for Safety and Health at Work (EU-OSHA) is developing as part of their Healthy Workplaces Campaign 2016–2017 an online e-guide entitled: ‘Healthy Workplaces for all Ages’ (project title: ‘Development of an online multilingual e-guide to support companies in occupational safety and health (OSH) management in the context of an ageing workforce’ – Open Tender Procedure No. EUOSHA-PRU/2014/P-3 – https://www.healthy-workplaces.eu/). The e-guide will support workers, employers, Human Resources managers, and OSH professionals to address ageing at work and to promote the health and safety of older workers, and will consist of national versions that are adapted to the context and languages of EU countries.

As part of the development phase of the national version of the e-guide for Belgium, we explored employers’ views on sustainable employability of older workers and their needs. The aims of our study are to explore how employers address ageing at the workplace, determine which OSH difficulties and barriers concerning worker ageing the companies are confronted with, and understand how companies promote sustainable employability (including CLA 104). Insight into these topics is important for adapting guidelines to current practices and needs.

## Methods

In the preparation phase of the master version of the e-guide, Milieu Ltd (coordinator of the EU-project) collaborated with Mensura Occupational Health Services (OHS) in order to reach Belgian employers and to assess their needs regarding sustainable work and how to promote employability among older workers. This cross-sectional study of Belgian companies was conducted between April 21, 2015 and April 29, 2015. A questionnaire was developed by a team of OSH researchers. The questionnaire consisted of 13 items: four items identify general characteristics (the function of the respondent, sector of activities, number of workers, and percentage of workers ≥55 years-old); five questions about the importance, needs, and concerns about sustainable employability in the company; and four questions concerning the knowledge and needs about sustainable employability policy. An-e-mail with an invitation to complete the online questionnaire was sent to the management of private-sector companies (*n* = 22,084) affiliated with Mensura OHS in Belgium. After three days, a reminder was sent.

## Results

### General characteristics

Ten percent of recipients (*n* = 2133) opened the e-mail, and within this group, 37.0 % (*n* = 790) completed the questionnaire (convenience sample). Respondents comprised 44.3 % directors, 23.7 % prevention advisors, 14.9 % human resources managers, and 17.2 % others. The most common sectors were: construction industry (21.9 %), other services (16.9 %), industry (13.5 %), human health and social work activities (9.2 %), and wholesale and retail trade and repair of motor vehicles and motorcycles (8.4 %). The numbers of workers in the companies were: 1–9 (38.4 %), 10–19 (16.7 %), 20–49 (16.7 %), 50–249 (19.7 %), 250–499 (4.3 %), and ≥500 (4.2 %). Most companies had less than 20 % of workers ≥55 years old (70.5 %); 24.8 % reported having 20 − 50 % of their workers ≥55 years old, and 4.7 % had >50 % workers ≥55 years old.

### Sustainable employability

Sustainable employability played a role (i.e. having special attention for the sustainable employability of workers ≥55 years old by e.g. having a policy, relevant initiatives or programmes) in most of the companies (89.4 %). Most of the companies did not have an active policy or initiative (69.3 %). Table 1 shows the reported topics concerning health and well-being of workers ≥55 years old that require special attention in the companies and the reported complaints of workers ≥55 years old.

**Table 1:** Topics concerning health and well-being of workers ≥55 years that require special attention and reported complaints of workers ≥55 years

	n	%
Topics concerning health and well-being of workers ≥55 years old that require special attention		
Keep workers ≥55 years old motivated and involved	309	29.6 %
Adapting work(place) to the (health) needs of workers ≥55 years old	269	25.7 %
Supporting workers ≥55 years old to maintain a good work-life balance	167	16.0 %
Prevention of illness or accidents in workers ≥55 years old	111	10.6 %
Identifying risks for workers ≥55 years old	96	9.2 %
Dealing with chronic or long-term diseases in workers ≥55 years old	93	8.9 %
Reported complaints of workers ≥55 years old		
Work-related health problems	255	30.7 %
Stress	212	25.5 %
Agreements on work and type of work	142	17.1 %
Work-life balance	122	14.7 %
Career development and/or training	76	9.2 %
Complaints related to safety	9	1.1 %
Discrimination	7	0.8 %
Harassment at work (bullying, violence, and sexual harassment)	7	0.8 %

Eighteen percent of the respondents experienced difficulties promoting sustainable employability of older workers. The reported difficulties were: lack of financial resources (23.5 %, *n* = 42), lack of know-how (22.3 %, *n* = 40), lack of interest of other managers to invest in sustainable employability (21.8 %, *n* = 39), lack of time (19.6 %, *n* = 35), and lack of participation of workers in relevant initiatives (12.8 %, *n* = 23).

Topics reported to be difficult to discuss with workers ≥55 years old were: the general ageing process and the impact on work (23.8 %, *n* = 118), stress (12.7 %, *n* = 63), long-term and chronic diseases (12.5 %, *n* = 62), flexible working hours (11.1 %, *n* = 55), career development (10.7 %, *n* = 53), general health problems (9.7 %, *n* = 48), pension issues (9.3 %, *n* = 46), work-life balance (7.3 %, *n* = 36), and disability (2.8 %, *n* = 14).

### Collective labour agreement 104 (CLA 104)

Almost half of the respondents (*n* = 380; 47.9 %) stated they do not know that since 01/01/2013, for companies in the private sector with >20 workers, it is mandatory to have a concrete action plan to promote the employment of workers ≥45 years old. Of the respondents whose company is required to develop a CLA 104 policy (>20 workers): 47.2 % (*n* = 180) had one, 34.4 % (*n* = 131) will develop this in the short term, and 18.4 % (*n* = 70) did not intend to do this.

Main focuses of the developed or planned CLA 104 policy were reported as: valuing the accumulated knowledge and experience (16.6 %, *n* = 333), ergonomic measures (13.8 %, *n* = 276), age-related reduction in working hours (9.8 %, *n* = 196), recruitment (8.6 %, *n* = 173), training (8.4 %, *n* = 169), psychosocial measures (7.8 %, *n* = 156), job matching (6.5 %, *n* = 130), age-related flexible work schedules (5.2 %, *n* = 104), sick leave policy (5.0 %, *n* = 100), spontaneous consultation with the OSH physician (4.8 %, *n* = 97), health promotion (4.2 %, *n* = 85), check-up examinations (4.0 %, *n* = 81), and others (5.2 %, *n* = 105).

## Discussion

Our study showed that, in most Belgian companies, sustainable employability of ageing workers is important; however, most companies do not yet have an active policy or initiative on this issue. These results underline the need for both Belgian and European support for employers to address an ageing workforce.

The annual report of the Experience Fund [4] showed that, despite the trend of an increasing number of applications, the Fund remains underutilized. Our study supports this finding. Based on the own study results and the annual report of the Experience Fund [4], we recommend that the Belgian government more extensively promotes the Experience Fund.

The results of this study will be used for the further development of the EU e-guide, and will ensure that it will: a) further increase employer awareness about the importance of implementing an active sustainable employability policy to prolong working life and productivity and b) include tools to address work-related health problems and stress, motivation, and workplace adaptation to the (health) requirements of workers aged ≥55 years. Similar surveys were conducted in all other EU countries and in the European Free Trade Association states (Norway, Iceland and Switzerland), to assess country-specific needs of employers concerning older-worker OSH issues. Country-specific surveys are important because a previous study showed that attitudes and actions of employers regarding the position of older workers in the EU vary by country [5].

This study has some limitations to be considered. Firstly, despite that survey answers were collected online and anonymously, provision of socially desirable answers cannot be excluded. Secondly, this study had a cross-sectional design and therefore only provides descriptive results. Thirdly, it is plausible that particularly respondents who are affiliated with the research topic and already have special attention for sustainable employability in their company, completed the questionnaire. Presumably, this has influenced the results. Fourthly, despite sending a reminder, the response was rather low. Some explanations could be provided. First, the period of data collection was rather short (between April 21, 2015 and April 29, 2015). Second, most of the e-mails were sent to general e-mail addresses of the companies and not directly to the managers of the companies because these were mostly unavailable.

Nonetheless, this report details important new findings that may enhance both company and employee experiences with an ageing workforce**.** Most studies on sustainable employability of older workers have been conducted from the economic, social security, health, and disability management perspectives. This study filled the knowledge gap by assessing employers' views and needs regarding employability of older workers in Belgium.

## Conclusion

Employers are particularly looking for tools and information about how to keep workers ≥55 years old motivated and involved, adapt work nature and setting for older workers' health needs, and support an ageing workforce to maintain a good work-life balance. Addressing these topics in the country-specific e-guide may facilitate widespread implementation of sustainable employability approaches in Belgium.
